# Who Gets More Trust—AI or Humans, and Why? A Cross-Cultural Analysis of AI and Interpersonal Trust

**DOI:** 10.3390/bs16030320

**Published:** 2026-02-26

**Authors:** Hui Zhang, Yiming Jing, Ruolei Gu

**Affiliations:** 1 State Key Laboratory of Cognitive Science and Mental Health, Institute of Psychology, Chinese Academy of Sciences, Beijing 100101, China; zhangh@psych.ac.cn; 2 Department of Psychology, University of Chinese Academy of Sciences, Beijing 100049, China

**Keywords:** AI trust, interpersonal trust, deception experience, risk propensity, trust propensity, cross-cultural differences, embodiment

## Abstract

As artificial intelligence (AI) systems become increasingly embedded in social contexts, understanding how individuals develop trust in AI relative to humans is critical. This study investigates the relative levels of trust in AI agents (embodied and disembodied) versus human social targets (intimate, intermediate, and distant groups), the psychological mechanisms underlying these trust patterns, and the potential cross-cultural differences between China and U.S. Moderated mediation models were tested to gain insights into how deception experience may affect trust via risk and trust propensity, with perceived honesty norms moderating the mediator-to-outcome pathways. Across both cultures, a consistent trust hierarchy emerged—AI was trusted less than close others but more than distant others. It is likely that, in China, embodied AI was evaluated through interpersonal trust schemas, while in the United States, AI was treated largely as a functional tool regardless of embodiment. Together, these findings clarify both the structure and the processes of AI trust, advancing theoretical debates over whether AI trust mirrors interpersonal trust and offering practical insights for designing culturally adaptive, trustworthy AI systems.

## 1. Introduction

Trust has long been regarded as a foundational element of social relationships, playing a central role not only in interpersonal interactions but increasingly in emerging forms of human-AI interaction (Simpson & Vieth, 2021). As AI systems become embedded in decision-making across domains—from healthcare and finance to education and social life—understanding how humans develop trust in AI has become increasingly vital (Brauner et al., 2024; Kaplan et al., 2023; M. K. Lee, 2018). Studies in human-AI interaction indicate that trust calibration plays a key role in system safety, user compliance, and technology uptake (Lewis et al., 2018; Tahmasbi et al., 2025). Under-trusting may lead to underutilization of beneficial automation, while over-trusting may foster automation complacency and overreliance (J. D. Lee & See, 2004).

Crucially, addressing trust miscalibration requires understanding not only how humans evaluate AI in isolation, but also how they compare it with the traditional benchmark of reliance: human judgment (Dietvorst et al., 2015; Dijkstra et al., 1998). Accordingly, a growing body of research examines the conditions under which individuals place greater trust in AI systems versus human agents (Sanders et al., 2019). A critical yet underexplored question is whether trust in AI represents a direct extension of interpersonal trust—either in magnitude or in underlying mechanisms—or whether it constitutes a qualitatively distinct process (de Visser et al., 2016; Sanders et al., 2019). If the two forms of trust are continuous, individuals with higher interpersonal trust should also exhibit greater trust in AI, and classical models of interpersonal trust—such as those emphasizing risk and trust propensity—should generalize to AI contexts (Huang & Bashir, 2017; Kraus et al., 2021). Alternatively, if trust in AI partly reflects dissatisfaction with human judgment, individuals low in interpersonal trust may place greater reliance on AI, particularly in contexts perceived as vulnerable to human bias or error (Molina & Sundar, 2022). Clarifying this relationship is theoretically important for understanding the applicability of classical trust models based on human interaction to nonhuman agents, and practically relevant for anticipating patterns of AI user adoption and reliance (Jain et al., 2023; Meng & Dai, 2021; Schaefer, 2013).

Despite the importance of understanding the relationship between trust in AI and trust in humans, few studies have examined this association directly, and even fewer have considered how it may vary across cultural contexts and types of trust (Chien et al., 2018; Duan et al., 2019; Haring et al., 2014). Yet, cultural values significantly shape how people perceive and evaluate AI agents (Castelo, 2024; Li et al., 2010). Prior research indicates that cultures differ in whether AI is primarily construed as an impartial, rule-based tool or anthropomorphized as an agent endowed with emotional or moral qualities, resulting in divergent trust dynamics (Dang & Liu, 2021; Haring et al., 2014; Wang et al., 2010). A cross-cultural comparison therefore provides a critical opportunity to examine whether trust in AI is grounded in the same psychological mechanisms as interpersonal trust, or whether these mechanisms are culturally contingent. Moreover, both the type of AI and the type of interpersonal trust may influence the degree of similarity between AI trust and human trust (Clark et al., 2019). Specifically, trust in AI is not monolithic; rather, it implies a dynamic alignment with different types of interpersonal trust. In some cases, it may mirror the reliance on close confidants, whereas in others, it parallels the cautious trust extended to unfamiliar agents (Brandtzaeg et al., 2022; Clark et al., 2019).

Trust does not emerge spontaneously but develops through dynamic interactions shaped by prior experiences (Hoff & Bashir, 2015; Rempel et al., 1985). These experiences influence trust primarily through underlying psychological processes rather than directly determining behavior (Falcone & Castelfranchi, 2004; Schilke et al., 2021). From the perspectives of instrumentalism and self-identity, risk propensity and trust propensity are two central factors influencing trust development (Dunning et al., 2014; Yamagishi et al., 2015). These individual-level psychological processes are further embedded within broader social contexts and can be moderated by social-level factors, such as perceived norms (Weber et al., 2002).

Building on this framework, we conceptualize trust formation as jointly driven by individual dispositions and social norms. On the individual side, we focus on risk propensity and trust propensity, which represent core pathways through which personal experiences shape the intention to trust (Dunning et al., 2014; Yamagishi et al., 2015). On the social side, because trust is not only an individual decision but also a socially regulated behavior, we highlight perceived honesty norms as a contextual factor that shapes how dispositions translate into trust (Gouldner, 1960; W. Johnson, 2013). Thus, our model integrates mediation (to explain how experiences influence trust) and moderation (to explain when these influences are stronger or weaker), providing a more comprehensive account of trust formation.

Building on these insights, the present study investigates both the trust patterns (who is trusted more—AI or humans) and the underlying psychological mechanisms (how AI trust is formed), examining how these processes converge or diverge across cultures. Specifically, we address two research questions:

Research Question 1: Is trust in AI psychologically continuous with interpersonal trust, or does it constitute a distinct form of trust? Specifically, which forms of interpersonal trust (intimate, intermediate, distant) most closely align with AI trust, and does this alignment differ between Chinese and American cultural contexts?

Research Question 2: Do the psychological mechanisms underlying trust formation—namely deception experience, risk propensity, trust propensity, and perceived honesty norms—operate differently for AI trust versus interpersonal trust, and are these mechanism-level differences culturally contingent?

Taken together, the present study makes two complementary contributions to the literature on trust in AI. First, at a structural level, we map the relative position of AI trust within a broader hierarchy of interpersonal trust, comparing trust in AI with trust in kin, intimate, intermediate, and distant social targets across cultural contexts. This allows us to clarify whether trust in AI mirrors, exceeds, or diverges from trust in different categories of human relationships. Second, at a process level, we unpack the psychological mechanisms underlying these trust patterns by examining how prior experiences, individual dispositions (risk propensity and trust propensity), and social norms jointly shape trust in AI and in humans. By integrating trust hierarchies with trust formation processes in a cross-cultural framework, this study advances a more comprehensive understanding of how and why people place trust in AI relative to human others.

## 2. Research Model and Hypotheses Development

### 2.1. The Relationship Between AI Trust and Interpersonal Trust

Two prominent theoretical perspectives on human–AI interaction offer contrasting predictions about the relationship between interpersonal trust and AI trust (de Visser et al., 2016; Nass et al., 1994). First, the Computers Are Social Actors (CASA) hypothesis posits that computers are perceived as social actors, leading humans to attribute anthropomorphic characteristics—such as gender and emotion—to AI and interact with it in human-like (K. M. Lee et al., 2006; Nass et al., 1994). Empirical evidence supports this perspective by showing that trust in AI systems often parallels trust in humans, potentially due to the activation of interpersonal cognitive scripts (Alarcon et al., 2021; de Visser et al., 2016). In contrast to the CASA model, the Unique Agent Hypothesis (UAH) argues that trust in AI and interpersonal trust are psychologically distinct processes. According to this view, human mind utilizes separate interaction schemas for humans and artificial agents (de Visser et al., 2016; Madhavan & Wiegmann, 2007). The differences between AI trust and interpersonal trust, proposed by the UAH, can be reflected in phenomena such as automation bias—an over-reliance on AI even when it is incorrect—and algorithm aversion—a tendency to prefer human advice over more accurate algorithmic recommendations (Dietvorst et al., 2015; Dijkstra et al., 1998; Manzey et al., 2012). Recent empirical findings further illustrate the nuanced interplay between these two perspectives (Berger et al., 2021; Maehigashi, 2022). For example, Berger et al. (2021) found that trust in unfamiliar human and algorithmic advisors is comparable, providing support for CASA. However, when familiarity was introduced, participants trusted familiar human advisors more than familiar algorithmic ones, aligning with UAH (Berger et al., 2021). Similarly, Maehigashi (2022) found that trust in robots partially aligns with interpersonal trust in subjective emotion recognition tasks (supporting CASA), but surpasses interpersonal trust in objective computational tasks (supporting UAH). Moreover, Molina and Sundar (2022) found that individuals low in interpersonal trust are more likely to trust AI for its perceived objectivity and accuracy—a finding that favors UAH over CASA.

In short, prior studies yield heterogeneous findings as to whether CASA or UAH better explains AI trust. We contend that these inconsistencies may stem from three critical yet often overlooked factors: (1) the cultural context in which trust is formed, (2) the specific form of interpersonal trust under consideration, and (3) the embodiment of AI (Biswas & Murray, 2025; Brandtzaeg et al., 2022; Zhang & Lee, 2024).

First, in Western societies, shaped by philosophical and religious traditions that position humans as uniquely endowed with mental privilege, people are generally more skeptical of anthropomorphizing AI and adopt a primarily functional view of robots (Haring et al., 2014). In contrast, East Asian cultures are more likely to perceive robots as capable of offering social and emotional services (Dang & Liu, 2021; Folk et al., 2025; Haring et al., 2014; H. R. Lee & Sabanović, 2014). Evidence from mass media and cultural narratives also suggests systematic cultural differences in how AI and robots are socially framed (Haring et al., 2014). American films often depict robots as dangerous technologies or uncontrollable threats, whereas in many East Asian cultural contexts robots are more frequently portrayed as helpers or heroes (Haring et al., 2014). Broader cultural value dimensions shape preferences for interpersonal versus algorithmic decision-making (Mahmud et al., 2022). Research grounded in the individualism–collectivism framework suggests that individuals in more individualistic cultures tend to rely on personal judgment, whereas those in collectivistic cultures may be more inclined to rely on algorithmic or system-based decisions (Mahmud et al., 2022). Surveys show that compared to Americans, individuals in developing countries such as China report greater trust in and acceptance of AI robots (Folk et al., 2025; Gillespie et al., 2023). Relatedly, cross-national research suggests that people from less corrupt regions prefer human decision-makers over algorithms, whereas participants from more corrupt countries show greater preference for algorithms (Castelo, 2024). Taken together, these findings suggest that cultural context may fundamentally shape how trust in AI is positioned relative to interpersonal trust (Dang & Liu, 2021). As prototypical representatives of individualistic and collectivistic cultural systems, the United States and China therefore provide a theoretically informative contrast for examining where human-AI trust is situated within the broader landscape of interpersonal trust, and how such trust is formed and differentiated across cultural contexts.

Second, the extent to which AI trust mirrors interpersonal trust may vary depending on which kind of interpersonal trust is considered. Interpersonal trust is commonly categorized into generalized trust—broad trust in others—and particularized trust, which refers to trust in specific, familiar groups such as family, friends, neighbors, or institutions (Ma, 2024; Schilke et al., 2021). A key determinant of particularized trust is relational distance, or the perceived social closeness between the trustor and trustee. This can be further divided into three levels: intimate, intermediate, and distant group trust (Jing et al., 2020; Jones, 2022). In our operationalization, distant group trust primarily captures trust toward strangers and thus conceptually overlaps with the core notion of generalized trust, whereas intimate and intermediate group trust fall within the broader category of particularized trust (Jing et al., 2020). Some studies suggest that individuals may form emotionally intimate bonds with AI, perceiving conversational agents as friends, family members, or even romantic partners (Brandtzaeg et al., 2022; Gao et al., 2018). Others find that robots are more often seen as strangers or utilitarian tools (Clark et al., 2019; Tistelgren, 2018).

Finally, embodiment constitutes another critical factor. Though definitions vary, in AI research embodiment generally refers to the integration of sensorimotor systems with environmental interaction, enabling situated intelligence (Duffy & Joue, 2000; Hellström et al., 2024; Jamison et al., 2008; McGregor, 2023). Embodied AI agents can perceive and act within physical space, unlike disembodied systems such as large language models (LLMs), which operate primarily through pattern recognition on vast textual or audiovisual datasets (Savva et al., 2019). Previous research shows that embodiment enhances the perception of AI agents as social actors, often eliciting higher trust, engagement, and perceived fairness compared to disembodied counterparts (Biswas & Murray, 2025). In our opinion, the degree to which such social cues translate into trust may be culturally contingent (Dang & Liu, 2021). East Asian cultures, which more readily attribute social and emotional affordances to technology, may be particularly responsive to the anthropomorphic signals provided by embodiment (Folk et al., 2025; Haring et al., 2014; H. R. Lee & Sabanović, 2014).

Accordingly, we propose that in the Chinese cultural context, embodiment will amplify AI trust to a greater degree than in the American context (Liu et al., 2024). Meanwhile, given the limited direct research on cross-cultural differences in trust toward disembodied AI versus interpersonal targets, we refrain from making a specific hypothesis on this point:

**H1a.**  *In the Chinese sample, trust in embodied AI would more closely resemble intimate or intermediate group trust*.

**H1b.**  *In the American sample, trust in embodied AI would more closely resemble distant group trust*.

### 2.2. The Moderated Mediation Model of AI Trust and Interpersonal Trust

Even if individuals exhibit similar levels of trust toward other people and AI agents, this does not necessarily imply that the two forms of trust share the same underlying psychological mechanisms. To address this, we examine how trust is formed in human–AI versus interpersonal contexts, aiming to further clarify the distinctions between the CASA and UAH theories.

#### 2.2.1. The Influence of Deception Experience on AI Trust and Interpersonal Trust

Trust evolves with experience: positive interactions tend to foster trust, whereas negative experiences may undermine it (Falcone & Castelfranchi, 2004; Schilke et al., 2021). Notably, negative experiences often exert a stronger and more dominant influence than positive ones (Cacioppo et al., 2014; Rozin & Royzman, 2001). Among negative experiences, deception plays a particularly detrimental role in trust formation (Peterson, 1996). Deception experience involves two key dimensions: frequency and consequences (Tyler et al., 2006). That is to say, both how often deception occurs and its qualitative nature influence the extent to which individuals develop negative perceptions of the deceiver (Ortmann & Hertwig, 2002; Tyler et al., 2006). For example, participants who have previously experienced deception are more likely to question the experimenter’s trustworthiness, reduce their willingness to participate in future experiments, and exhibit diminished performance when they do (Cook et al., 1970; Jamison et al., 2008). This dynamic applies not only to interpersonal trust but also to human-AI trust. That is, participants who perceive robots as dishonest tend to report significantly reduced trust (Rogers et al., 2023; Sebo et al., 2019), suggesting that prior deception undermines trust regardless of whether the agent is human or artificial.

Importantly, the impact of deception experience on trust may vary across cultures (Nisbett et al., 2001). Chinese individuals, who tend to adopt holistic thinking and rely more on contextual and experiential knowledge, may be more sensitive to past experiences of trust violations (Nisbett et al., 2001). By contrast, Westerners, characterized by more analytic thinking, emphasize object attributes and rule-based reasoning (Nisbett et al., 2001), potentially reducing the weight of prior experiences in trust formation. Cultural time orientation further shapes this process: Chinese individuals place greater weight on past events (Guo et al., 2012; Guo & Spina, 2019), whereas Americans tend to prioritize the future over the past (Caruso et al., 2008). East Asian cultures, characterized by collectivism, strong uncertainty avoidance, and high long-term orientation, encourage trust evaluations that incorporate future stability of performance (S.-G. Lee et al., 2013). For example, Korean participants were more likely than Americans to assume that performance flaws would persist over time, thereby lowering trust in robots based on competence and integrity judgments (Zhang & Lee, 2024).

Based on this, we propose the following hypothesis:

**H2.**  *The association between deception experience and AI trust is expected to vary across cultural contexts, with a more salient negative pattern in the Chinese sample*.

#### 2.2.2. The Mediating Role of Risk Propensity and Trust Propensity on AI Trust and Interpersonal Trust

Past experiences influence trust by shaping individuals’ psychological dispositions. According to widely accepted definitions, trust involves two key dimensions: the evaluation of others’ trustworthiness and the individual’s willingness to accept vulnerability (J. D. Lee & See, 2004; Mayer et al., 1995). The former dimension relates to one’s risk propensity, while the latter corresponds to trust propensity (Dunning et al., 2014; Yamagishi et al., 2015).

Risk propensity. From the perspectives of instrumentalism and consequentialism, trust serves as a means for individuals to achieve their goals (Dunning et al., 2014). Individual differences in trust-related decision-making can thus be predicted by risk propensity—the tendency to either seek or avoid risk in decision-making contexts (Sitkin & Pablo, 1992). According to the risk-return framework of decision-making, risk propensity is determined by the perceived balance between benefits and risks, moderated by individuals’ risk attitudes, typically expressed as risk aversion (Blais & Weber, 2006; Sitkin & Pablo, 1992; Weber et al., 2002). Previous studies have found that interpersonal trust is negatively associated with risk perception and positively associated with risk propensity (Alarcon & Jessup, 2023; Ilhamalimy & Ali, 2021). However, empirical research on the relationship between AI trust and risk propensity remains limited. Some findings suggest that individuals with lower risk-seeking tendencies tend to exhibit greater trust in automated systems (Ferronato & Bashir, 2020).

Because perceived risks and benefits vary across domains, risk propensity demonstrates domain specificity (Weber et al., 2002). In their study, Weber and colleagues (2002) identified five domains in which risk-taking behavior varies: ethical, financial, health/safety, social, and recreational. Among these, financial and social risk are most relevant to the present study. Financial risk refers to concerns about potential material or monetary gambling and investment, which frequently arise both in interpersonal exchanges (e.g., trust in financial honesty) and in AI-mediated decisions (Blais & Weber, 2006; Weber et al., 2002). Meanwhile, social risk involves potential threats to reputation, relationships, or social standing—particularly salient when AI is perceived as a social actor (Xing et al., 2025). Other domains, such as health/safety or recreational risk, while important in certain contexts, are less central to the comparison between interpersonal and sociotechnical trust considered here.

Importantly, the physical embodiment of technologies such as autonomous vehicles or industrial robots introduces unique risk profiles, extending beyond financial risks to physical safety and social norm violations (Xing et al., 2025). These additional risk dimensions become especially salient when AI is perceived not merely as a tool but as a social actor. Moreover, as noted above, cultural context modulates this perception: whereas Americans are more likely to adopt a functional view of AI, Chinese individuals tend attribute social and emotional affordances to it (Dang & Liu, 2021; Haring et al., 2014; H. R. Lee & Sabanović, 2014). Thus, we hypothesize:

**H3a.**  *In the Chinese sample, the relationship between deception experience and AI trust is expected to be mediated by risk propensity, with social risk and financial risk representing distinct pathways depending on AI embodiment*.

**H3b.**  *In the American sample, financial risk is expected to play a more central mediating role in the relationship between deception experience and AI trust, regardless of AI embodiment*.

Trust propensity. Risk propensity does not fully explain interpersonal trust behavior (Dunning et al., 2014). From a self-identity perspective, trust propensity—the general tendency to view oneself as a trusting person and to act accordingly—is also worth noting (Yamagishi et al., 2015). Individuals with higher trust propensity are generally more willing to trust others (Alarcon et al., 2016; Alarcon & Jessup, 2023; Colquitt et al., 2007). Notably, trust propensity also positively predicts trust in AI technologies: those who are more inclined to trust other people are also more likely to trust automated systems as well (Aliasghari et al., 2021; Huang & Bashir, 2017; Rossi et al., 2018; Yi et al., 2023).

When both risk propensity and trust propensity are included in predictive models, trust propensity tends to exert a stronger influence on interpersonal trust (Dunning et al., 2014). However, few studies have directly compared these two predictors in the context of human-AI trust. As AI technologies become more advanced, they are increasingly perceived not merely as tools but as collaborators or teammates—especially in the case of embodied AI (Rix, 2022). For instance, people evaluate not only the competence of AI, but also its moral character and behaviors (Malle & Ullman, 2021). This suggests that AI trust may not rely solely on traditional risk assessments. Moreover, compared to disembodied AI, embodied AI tends to evoke a stronger sense of social presence, leading users to experience them as actual social actors (Jung & Lee, 2004; K. M. Lee et al., 2006).

As we mentioned above, due to philosophical, religious, and cognitive factors, Westerners find it more difficult to accept the anthropomorphism of robots and tend to adopt a predominantly functional perspective, viewing robots as assistants, whereas East Asians are more likely to expect robots to provide social services (Dang & Liu, 2021; Folk et al., 2025; Haring et al., 2014; H. R. Lee & Sabanović, 2014). Accordingly, we hypothesize that:

**H4a.**  *In the Chinese sample, individual differences in propensity are expected to play a mediating role in the association between deception experience and AI trust, with trust propensity and risk propensity representing distinct psychological pathways across AI types*.

**H4b.**  *In the American sample, risk propensity is expected to be more relevant than trust propensity in the association between deception experience and AI trust, regardless of AI embodiment*.

#### 2.2.3. The Moderating Role of Perceived Honesty Norms on AI Trust and Interpersonal Trust

Normative beliefs refer to an individual’s perception of whether significant others or respected social groups approve or disapprove of a given behavior (Ajzen & Fishbein, 2005). These beliefs contribute to perceived social pressure—or subjective norms—which, together with personal attitudes and perceived behavioral control, shape behavioral intentions and ultimately influence actual behavior (Weber et al., 2002). Trust is fundamentally a reciprocal process between two parties and is closely tied to the perceived trustworthiness of the target. According to the reciprocity theory, individuals tend to reciprocate in the way that they are treated by others (Gouldner, 1960; W. Johnson, 2013). Consequently, trust-related behavior is often guided by perceived honesty norms—that is, beliefs about how honest most people in society generally are (a form of descriptive norm).

Individuals in tight honesty cultures such as China perceive stronger social norms and show lower tolerance for deviance, whereas those in loose cultures such as the United States experience greater behavioral flexibility and weaker normative constraints (Gelfand et al., 2011). Tight cultural environments promote greater caution, impulse control, and self-monitoring, which may in turn constrain individuals’ willingness to place trust in others (Gelfand et al., 2011). Furthermore, as AI technologies increasingly function as collaborative agents, we propose that these social norms may extend to human-AI interactions—even though AI is not a genuinely cognitive or moral entity, this effect may be particularly salient in China and for embodied AI (Castelfranchi & Falcone, 1998). Based on this reasoning, we hypothesize that:

**H5.**  *Perceived honesty norms are expected to moderate the relationship between individual dispositions (risk propensity and trust propensity) and AI trust, such that the relative influence of these dispositions varies across cultural contexts and AI embodiment*.

**Figure 1:**
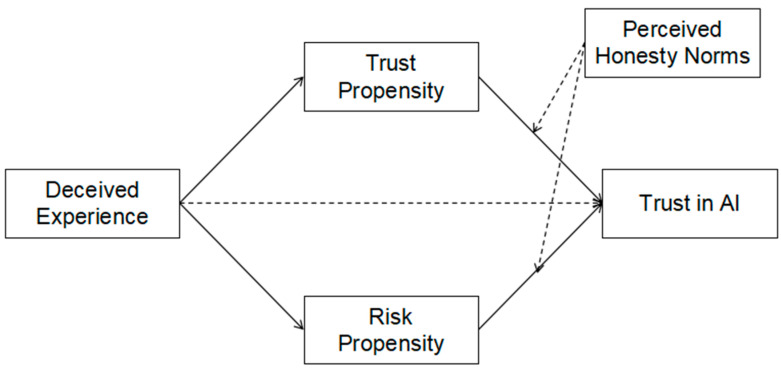
Moderated mediation model of AI trust (Dashed lines indicate effects observed exclusively in the Chinese sample).

Accordingly, a moderated mediation model was constructed, as illustrated in Figure 1. Specifically, the model examines whether the proposed pathway operates differently for interpersonal trust versus human-AI trust, and whether the strength or structure of this pathway further varies across cultural groups.

## 3. Method

### 3.1. Sample

The required sample size was determined using G*Power 3.1 based on three separate power analyses to meet the study’s analytical needs (Faul et al., 2007). First, assuming a medium effect size (*ρ* = 0.30) for correlation analyses, a minimum of 111 participants was required. Second, for paired-samples *t*-tests with a medium effect size (*dz* = 0.50), at least 45 participants were needed. Third, multiple regression analyses with six predictors and a medium effect size (*f*^2^ = 0.15) indicated a minimum of 146 participants. In line with previous research (Eneizan et al., 2005; Sinha & Singh, 2022) and to ensure sufficient power despite potential dropouts or data errors, we recruited 577 individuals for the study (see below for details). This study was preregistered on the Open Science Framework (OSF; https://osf.io/m9xuk/overview?view_only=5569cb3d207b473a9de4670885720a99, accessed on 31 January 2026).

A cross-cultural sample of 300 Chinese and 300 American participants was recruited via Credamo (a Chinese online survey platform; https://www.credamo.com/, accessed on 15 April 2025) and Prolific (a U.K.-based research platform; https://www.prolific.co/, accessed on 15 April 2025), respectively. Given that Prolific is based in the UK, participant recruitment was restricted to individuals currently residing in the United States. To better understand the ethnic composition of our sample, we also collected self-reported nationality information. Of the American sample, 271 participants identified their nationality as American nationals, while 29 reported other nationalities (1 Asian, 1 Bangladeshi, 1 Brazilian, 2 British, 3 Chinese, 1 Congolese, 2 European, 1 French Canadian, 1 Hispanic, 1 Indian, 2 Latino/a, 3 Mexican, 2 Nigerian, 1 Philippines, 1 Polynesian, 1 Ukrainian, 4 White, 1 no answer). To ensure cultural consistency and reduce potential confounds stemming from cross-cultural variability, we re-contacted these 29 participants to clarify their residency status. Those who indicated they were only temporarily residing in the United States, as well as those who did not respond, were excluded from the final sample.

Chinese participants were collected in two waves: the first in September 2024 and the second in February 2025. American participants were recruited between 25 February and 10 March 2025. Compensation was provided in accordance with platform-specific ethical guidelines, with Chinese participants receiving 10 CNY (approximately 1.38 USD) and American participants 1.65 GBP (approximately 2.09 USD) upon task completion.

To ensure sample validity and comparability, individuals outside the age range of 18 to 50 were excluded, as this range is commonly regarded as representative of the general adult population in psychological research (M. D. Johnson et al., 2021). Additionally, participants not meeting the eligibility criteria for the American sample were removed. After these exclusions, the final analytical sample consisted of 289 Chinese and 288 American participants (*N* = 577). Table 1 provides an overview of 577 participants’ demographic and occupational characteristics. Among the Chinese participants, there were 118 men (40.8%) and 171 women (59.2%); among the American participants, 146 were men (50.7%) and 142 were women (49.3%). The mean age of Chinese participants was 28.84 years (*SD* = 5.32), and that of American participants was 27.86 years (*SD* = 4.70).

### 3.2. Measurement

The study assesses key constructs using validated scales from the existing literature and self-developed questionnaires (see below). All instruments were administered in parallel Chinese-English bilingual versions. Except for the self-developed questionnaire, which was originally created in Chinese, all other measures had established English versions. With the assistance of ChatGPT (GPT-4) (developed by OpenAI, https://chat.openai.com/, accessed on 15 April 2025), two bilingual researchers translated all materials from their original Chinese or English versions into the corresponding language and conducted back-translation to ensure linguistic and conceptual equivalence.

#### 3.2.1. Deception Experience Scale

Deception experience was assessed using a newly developed 2-item scale (Peterson, 1996; Tyler et al., 2006). The scale employed a dual-axis framework measuring (1) prevalence (“Have you ever been deceived or tricked in your life?”), and (2) perceived psychological impact (“To what extent has being deceived impacted you?”). This approach concurrently evaluates both the frequency and subjective salience of deceptive interactions.

#### 3.2.2. Risk Propensity Scale

Risk propensity was measured using items adapted from the Domain-Specific Risk-Taking (DOSPERT) Scale, assessing individuals’ risk propensity across two dimensions: social risk and financial risk (Weber et al., 2002). Responses were recorded on a 5-point Likert type scale ranging from 1 (very unlikely) to 5 (very likely). Psychometric evaluation demonstrated acceptable internal consistency across cultural groups, with Chinese participants showing Cronbach’s *α* coefficients of 0.73 for the social risk subscale and 0.74 for the financial risk subscale. American participants exhibited comparable reliability for financial risk (*α* = 0.83), though slightly reduced consistency for social risk assessments (*α* = 0.60). 

#### 3.2.3. Trust Propensity Scale

Trust propensity was assessed using the four-item Trust Preference Scale, which has demonstrated strong reliability and validity (Yamagishi et al., 2015), with Cronbach’s α coefficients of 0.72 for Chinese participants and 0.26 for American participants. Responses were recorded on a 5-point Likert scale ranging from 1 (strongly disagree) to 5 (strongly agree).

#### 3.2.4. Perceived Honesty Norms Scale

Perceived honesty norms were assessed using a self-developed four-item questionnaire, designed based on theoretical frameworks of cultural tightness-looseness, reciprocity, and social norm enforcement (Gelfand et al., 2011; Jacobson et al., 2015; Perugini et al., 2003). Items included statements such as: “People who resort to fraud and deception will be punished,” “Most people are honest and trustworthy,” “People who abide by the rules and uphold principles tend to suffer losses,” and “Good deeds will be rewarded.” Participants rated the extent to which each statement reflected their current social environment on a 5-point Likert scale, ranging from 1 (strongly inconsistent) to 5 (strongly consistent). The scale demonstrated Cronbach’s *α* coefficients of 0.78 for Chinese participants and 0.51 for American participants.

#### 3.2.5. Interpersonal Trust Scale

Building on the World Values Survey (WVS) Wave 5 scale (Welzel, 2010) and the social distance-based Interpersonal Trust Scale (Jing et al., 2020), we developed a measure of individuals’ trust in different social groups. Participants were asked to indicate their level of trust toward each specific target on a five-point Likert scale ranging from 1 (very untrustworthy) to 5 (very trustworthy). The measure included three interpersonal trust dimensions: (1) Intimate Group Trust, assessed by trust in family, familiar relatives and close friends; (2) Intermediate Group Trust, assessed by trust in unfamiliar relatives and ordinary friends; and (3) Distant Group Trust, assessed by trust in strangers. Importantly, each social target was assessed with a single, distinct item and was included in only one trust dimension, such that there were no overlapping items across subscales. Scores for each trust dimension were computed by summing participants’ trust ratings for the corresponding targets and dividing by the number of items, such that higher scores indicate greater trust toward that social group. The Intimate Group Trust scale demonstrated Cronbach’s *α* coefficients of 0.74 for Chinese participants and 0.72 for American participants. The Intermediate Group Trust scale demonstrated Cronbach’s *α* coefficients of 0.66 for Chinese participants and 0.38 for American participants.

#### 3.2.6. Artificial Intelligence Trust Scale

Given evidence that people perceive embodied AI (e.g., robots) and disembodied AI (e.g., large language models) differently (Kwak et al., 2013; K. M. Lee et al., 2006), we extended the Interpersonal Trust Scale described above by adding two items: “Embodied Artificial Intelligence” and “Disembodied Artificial Intelligence.” To ensure comprehension, illustrative examples were provided in the pre-experiment instructions.

#### 3.2.7. Demographic Information

Demographic information included participants’ age, biological and psychological gender, educational attainment, and subjective socioeconomic status (SES). SES was assessed using the MacArthur Scale of Subjective Social Status (Adler et al., 2000), in which participants rated their perceived familial social status on a 10-point ladder scale.

### 3.3. Procedures

Before beginning the experiment, participants provided informed consent and were introduced to the concepts of embodied and disembodied AI. These categories were defined based on the distinction between tangible physical hardware and virtual software agents. The experimental instruction stated: “Artificial intelligence (AI) can be either embodied (e.g., everyday household smart speakers, robotic vacuum cleaners, DJI drones, military robots) or disembodied (e.g., software systems, chatbots, Siri, Tmall Genie).” To minimize order effects, all questionnaires—including those on deception experience, risk propensity, trust propensity, perceived social norms, interpersonal trust, and AI trust—were presented in randomized order across participants. After completing all psychometric measures, participants filled out a demographic questionnaire.

### 3.4. Data Analysis

Data analyses were performed using R (Version 4.4.3; https://cran.r-project.org/bin/windows/base/, accessed on 15 April 2025), encompassing descriptive statistics, difference tests, and regression analyses. Results are reported in accordance with APA 7th edition guidelines, including key statistical indices such as mean (*M*), standard deviation (*SD*), *t*-values, and *p*-values. A p-value below 0.05 was considered statistically significant.

To test the proposed moderated mediation models, we employed the PROCESS framework implemented in R using the BruceR package (Bao, 2025). Specifically, deception experience was specified as the independent variable (X), trust as the dependent variable (Y), risk propensity and trust propensity as parallel mediators (M), and perceived honesty norms as a moderator (W) of the mediator–outcome paths. Age, biological sex, education level, and subjective socioeconomic status were included as covariates in all models. Indirect effects were estimated using a nonparametric bootstrap procedure with 5000 resamples. Bias-corrected 95% confidence intervals (CIs) were computed for all conditional indirect effects. An indirect effect was considered statistically significant when the corresponding confidence interval did not include zero.

Some scales (Deception Experience Scale, Risk Propensity Scale, Trust Propensity Scale, Perceived Honesty Norms Scale, and Interpersonal Trust Scale) included a “Hard to say” response option, intended to identify potentially ambiguous items. Fewer than 10% of participants selected this option for any given item, indicating acceptable item clarity. To address these responses, three handling strategies were tested: treating them as missing data, replacing them with the scale midpoint, or imputing participants’ mean scores. All approaches yielded consistent findings. As the findings were robust to different treatments, mean substitution was used in subsequent analyses.

## 4. Results

### 4.1. Question 1: The Relationship Between AI and Interpersonal Trust

#### 4.1.1. Correlation Analyses of AI Trust and Interpersonal Trust

Pearson’s correlation analyses were conducted to examine the associations between AI trust indices (embodied AI and disembodied AI) and various types of interpersonal trust (intimate group, intermediate group, and distant group) (Table 2). AI trust was positively correlated with all types of interpersonal trust in both cultural groups (Chinese: *rs* ≥ 0.15, *ps* < 0.05; American: *rs* ≥ 0.17, *ps* < 0.01).

#### 4.1.2. Comparative Analysis of AI and Interpersonal Trust Levels

**Figure 2:**
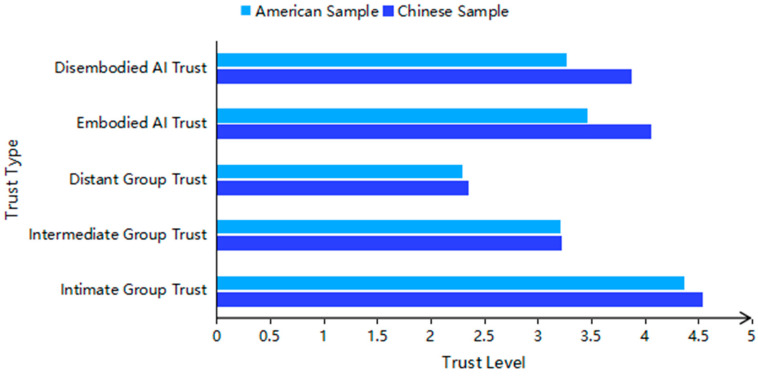
Trust Hierarchy in Chinese and American Sample.

To test H1, paired-sample *t*-tests were conducted comparing the two dimensions of AI trust (embodied and disembodied) with the four dimensions of interpersonal trust (intimate group, intermediate group, and distant group) (Figure 2).

The results for Chinese participants did not support H1a, instead revealing the following patterns: (1) Trust in embodied AI was significantly lower than intimate group trust, *t* (288) = −11.86, *p* < 0.001, Cohen’s *d* = −0.70, and significantly higher than both intermediate group trust, *t* (288) = 17.74, *p* < 0.001, Cohen’s *d* = 1.04, and distant group trust, *t* (288) = 29.24, *p* < 0.001, Cohen’s *d* = 1.72. (2) Trust in disembodied AI was significantly lower than intimate group trust, *t* (288) = −15.77, *p* < 0.001, Cohen’s *d* = −0.93. Conversely, it was significantly higher than both intermediate group trust, *t* (288) = 13.04, *p* < 0.001, Cohen’s *d* = 0.77, and distant group trust, *t* (288) = 24.54, *p* < 0.001, Cohen’s *d* = 1.44.

The results for American participants did not support H1b, instead revealing the following patterns: (1) Trust in embodied AI was significantly lower than intimate group trust, *t* (287) = −13.85, *p* < 0.001, Cohen’s *d* = −0.82. Conversely, it was significantly higher than both intermediate group trust, *t* (287) = 3.50, *p* < 0.001, Cohen’s *d* = 0.21, and distant group trust, *t* (287) = 14.80, *p* < 0.001, Cohen’s *d* = 0.87. (2) Trust in disembodied AI was significantly lower than intimate group trust, *t* (287) = −16.52, *p* < 0.001, Cohen’s *d* = −0.97. It did not significantly differ from intermediate group trust, *t* (287) = 0.74, *p* = 0.46, Cohen’s *d* = 0.04, but was significantly higher than distant group trust, *t* (287) = 12.22, *p* < 0.001, Cohen’s *d* = 0.72.

### 4.2. Question 2: Differential Impact Patterns of Predictors on AI Trust and Interpersonal Trust

To test our hypotheses (H2–H5), we conducted three types of analyses: (1) mediation analyses examining the effects of different dimensions of risk propensity; (2) mediation analyses comparing the effects of overall risk propensity and trust propensity; and (3) moderated mediation analyses testing whether perceived honesty norms moderated the effects of risk propensity and trust propensity on trust.

#### 4.2.1. Differential Impact of Social Risk and Financial Risk on AI Trust and Interpersonal Trust

We employed Model 4 of the PROCESS macro to test direct and indirect effects with social risk and financial risk as mediators. First, H2 was supported (see Table 3 for details). In the Chinese sample, deception experience showed a significant negative association with trust in disembodied AI (*b* = −0.08, *p* < 0.01); however, no such association emerged in the American sample. Second, H3 was not directly supported. Specifically, in both the Chinese and American samples, neither social risk nor financial risk significantly mediated the relationship between deception experience and trust (including both AI trust and interpersonal trust). Moreover, there were no significant differences between the mediation effects of social risk and financial risk.

Interestingly, in the Chinese sample, for trust in embodied AI, social risk was significantly positively associated with trust (*b* = 0.03, *p* = 0.00), whereas financial risk was not significantly associated with trust (*b* = 0.02, *p* = 0.13); however, the difference between these associations were not significant (*b* = 0.00, *p* = 0.87), providing partial support for H3a. For trust in disembodied AI, social risk was not significantly associated with trust (*b* = 0.01, *p* = 0.31), whereas financial risk was significantly positively associated with trust (*b* = 0.05, *p* = 0.00); moreover, the association between financial risk and trust was significantly stronger than that of social risk (*b* = 0.04, *p* = 0.05), providing partial support for H3a.

In the American sample, for trust in embodied AI, social risk was not significantly associated with trust (*b* = −0.05, *p* = 0.62), whereas financial risk was significantly positively associated with trust (*b* = 0.41, *p* < 0.001); moreover, the association involving financial risk was significantly stronger than that involving social risk (*b* = 0.36, *p* < 0.001), providing partial support for H3b. For trust in disembodied AI, social risk was not significantly associated with trust (*b* = −0.14, *p* = 0.19), whereas financial risk was significantly positively associated with trust (*b* = 0.31, *p* < 0.001); however, the difference between these associations was not significant (*b* = 0.17, *p* = 0.10), providing partial support for H3b.

These findings suggest that across cultural contexts, different dimensions of risk propensity are differentially associated with trust in embodied versus disembodied AI.

#### 4.2.2. Differential Impact of Risk Propensity and Trust Propensity on AI Trust and Interpersonal Trust

We employed Model 4 of the PROCESS macro to test indirect effects of risk propensity and trust propensity. The results indicated that H4a was supported (Table 4).

In the Chinese sample, for trust in embodied AI, the indirect association via risk propensity was not significant (*ab* = −0.01, *p* = 0.17, 95% CI [−0.028, −0.001]), whereas the indirect association via trust propensity was significant (*ab* = −0.03, *p* < 0.001, 95% CI [−0.050, −0.017]), with the latter showing a stronger effect (*b* = 0.02, *p* = 0.05). For disembodied AI, neither the mediation effect of risk propensity (*ab* = −0.01, *p* = 0.11, 95% CI [−0.033, −0.001]) nor that of trust propensity (*ab* = −0.01, *p* = 0.27, 95% CI [−0.025, 0.005]) was significant, thus supporting for H4a. For interpersonal trust, the mediation effect of risk propensity was not significant across all types of trust. In contrast, the mediation effect of trust propensity was significant for all types of interpersonal trust (Table 4). Additionally, the mediation effects of trust propensity were significantly stronger than those of risk propensity. In short, we observed that in the Chinese sample, trust in embodied AI and interpersonal trust exhibited similar mediation patterns, whereas this was not the case for disembodied AI.

In the American sample, neither indirect association via risk propensity (embodied AI: *ab* = 0.01, *p* = 0.12, 95% CI [−0.003, 0.032]; disembodied AI: *ab* = 0.01, *p* = 0.20, 95% CI [−0.002, 0.020]) nor that of trust propensity (embodied AI: *ab* = −0.00, *p* = 0.81, 95% CI [−0.010, 0.007]; disembodied AI: *ab* = −0.00, *p* = 0.72, 95% CI [−0.010, 0.007]) was significant for AI trust, providing no support for H4b. For interpersonal trust, the mediation effect of risk propensity remained non-significant across all types of trust. Meanwhile, the mediation effect of trust propensity was marginally significant for all types of interpersonal trust; however, the differences between trust and risk propensity were not significant. These results suggest that for American participants, trust propensity functioned as a mediator only in the case of interpersonal trust, but not AI trust, while risk propensity exerted no significant effect in either domain.

#### 4.2.3. The Moderated Mediation Effects of Risk Propensity and Trust Propensity on AI and Interpersonal Trust

Using Model 14 in PROCESS macro, we examined moderated mediation effects of risk propensity and trust propensity on both AI and interpersonal trust, with perceived honesty norms moderating the path from the mediator to the outcome, H5 was supported.

In the Chinese sample, for embodied AI trust, perceived honesty norms moderated the associations between both risk propensity (*F*(1, 278) = 5.69, *p* = 0.02) and trust propensity (*F*(1, 278) = 6.19, *p* = 0.01) and trust. Specifically, a switching pattern was observed: the positive association between risk propensity and embodied AI trust was observed at low levels of perceived honesty norms (*b* = 0.03, *p* < 0.001), whereas the positive association between trust propensity and embodied AI trust was evident at high levels (*b* = 0.08, *p* < 0.001). For disembodied AI trust, perceived honesty norms moderated the association between risk propensity and trust (*F*(1, 278) = 13.70, *p* < 0.001), but did not moderate the association between trust propensity and trust (*F*(1, 278) = 2.51, *p* = 0.12) (see Table 5). For interpersonal trust, perceived honesty norms did not moderate the association between risk propensity and trust, but did moderate the association between trust propensity and trust (see Table 6).

In the American sample, for AI trust, perceived honesty norms did not moderate the association between risk propensity and trust (embodied AI: *F*(1, 277) = 0.58, *p* = 0.45; disembodied AI: *F*(1, 277) = 1.11, *p* = 0.29), but did moderate the association between trust propensity and trust (embodied AI: *F*(1, 277) = 7.51, *p* = 0.01; disembodied AI: *F*(1, 277) = 3.88, *p* = 0.05). For interpersonal trust, perceived honesty norms did not moderate the associations involving either risk propensity or trust propensity (see Table 6).

These findings suggest that perceived honesty norms are differentially associated with how individual dispositions relate to AI trust across cultural contexts. In the Chinese context, honesty norms appear to differentiate whether embodied versus disembodied AI trust is more closely associated with risk propensity or trust propensity. In contrast, in the American context, honesty norms are associated primarily with variation in the association between trust propensity and AI trust, with no comparable pattern observed for risk propensity.

### 4.3. Additional Analyses

Given the possibility that our instructional descriptions of embodied and disembodied AI in the initial data collection might have introduced ambiguity in participants’ perceptions, we included measures of participants’ usage frequency of different AI types in the second wave of the Chinese sample and in the U.S. sample. This allowed us to statistically account for potential familiarity effects when examining trust in embodied and disembodied AI.

After controlling for AI usage frequency, the results in the Chinese sample showed some changes. Specifically, deception experience was no longer significantly associated with either embodied AI trust (*b* = −0.03, *p* = 0.33) or disembodied AI trust (*b* = −0.01, *p* = 0.69), providing no support for Hypothesis 2a. Regarding the differential effects of social risk and financial risk on AI trust, financial risk remained significantly associated with disembodied AI trust (*b* = 0.21, *p* = 0.01), whereas neither social risk (*b* = 0.07, *p* = 0.41) nor financial risk (*b* = 0.11, *p* = 0.18) was significantly related to embodied AI trust. Thus, the hypothesized pattern that embodied AI trust would be more strongly guided by social risk was not supported in the Chinese subsample. In addition, analyses examining the moderating role of perceived honesty norms indicated that, for both embodied and disembodied AI, the interaction effects between perceived honesty norms and risk propensity (embodied AI: *F*(1, 134) = 0.40, *p* = 0.53; disembodied AI: *F*(1, 134) = 0.32, *p* = 0.58) or trust propensity (embodied AI: *F*(1, 134) = 2.22, *p* = 0.14; disembodied AI: *F*(1, 134) = 2.08, *p* = 0.15) were not statistically significant in the Chinese sample.

In contrast, in the U.S. sample, controlling for AI usage frequency did not alter any of the main results. All previously reported effects remained statistically unchanged, indicating a relatively high level of robustness in this sample. We note that the reduced sample size in the Chinese subsample included in these additional analyses (*n* = 146) may have limited statistical power and contributed to the observed discrepancies. Nonetheless, the stability of the findings in the U.S. sample provides support for the overall robustness of the core conclusions.

To further examine the relative position of AI trust within the broader trust landscape across cultural contexts, we additionally measured overall AI trust using the AI Trust Scale (Hoffman et al., 2023), which has demonstrated strong reliability and validity (Perrig et al., 2023). Results from the Chinese sample showed that overall AI trust was significantly lower than intimate group trust, *t* (288) = −19.68, *p* < 0.001, Cohen’s *d* = −1.16, but significantly higher than both intermediate group trust (*t* (288) = 15.42, *p* < 0.001, Cohen’s *d* = 0.91) and distant group trust (*t* (288) = 27.67, *p* < 0.001, Cohen’s *d* = 1.63). Overall AI trust was negatively associated with deception experience (*b* = −0.08, *p* < 0.001). Mediation analyses further indicated that social risk significantly mediated this relationship (*ab* = −0.02, *p* = 0.03, 95% CI [−0.032, −0.003]). In addition, both risk propensity (*ab* = −0.01, *p* = 0.065, 95% CI [−0.027, −0.002]) and trust propensity (*ab* = −0.03, *p* < 0.001, 95% CI [−0.052, −0.019]) showed significant indirect effects. Moderated mediation analyses revealed that perceived honesty norms significantly interacted with both risk propensity, *F*(1, 278) = 9.18, *p* < 0.01, and trust propensity, *F*(1, 278) = 28.47, *p* < 0.001. Simple effects analyses indicated that under low perceived honesty norms, risk propensity significantly predicted AI trust, whereas under high perceived honesty norms, trust propensity emerged as the stronger predictor.

Results from the American sample largely converged with those observed in the Chinese sample with respect to the relative positioning of overall AI trust within the broader trust landscape. However, several notable differences emerged in the process-level analyses. Specifically, although overall AI trust was again negatively associated with deception experience (*b* = −0.05, *p* < 0.05), neither social risk nor financial risk significantly mediated this relationship. Moreover, neither risk propensity nor trust propensity showed significant indirect effects in the American sample. Moderated mediation analyses further indicated that perceived honesty norms significantly interacted with trust propensity only, *F*(1, 277) = 6.44, *p* < 0.05.

Analyses of overall AI trust further illuminate cultural differences in trust formation processes. At the same time, the results show partial convergence between the findings for specific AI types (embodied vs. disembodied) and those for overall AI trust, suggesting that similar psychological mechanisms operate across different levels of AI abstraction, albeit with variation across dimensions.

## 5. Discussion

Overall, this study addressed two key questions through a cross-cultural lens (see Table 7): (1) What is the nature of the relationship between AI trust and interpersonal trust across cultures? (2) How does deception experience influence trust in different types of AI and interpersonal relationships, through the mediating roles of risk and trust propensity, and the moderating role of perceived honesty norms? And, how do these processes differ between cultural contexts?

First, in terms of trust levels, across both Chinese and American samples, trust in AI—whether embodied or disembodied—was consistently lower than trust in intimate groups but higher than trust in distant groups. This cross-cultural trust levels consistency suggests that both Chinese and American participants may perceive AI agents as moderately close social partners—neither fully integrated into inner social circles, nor as distant or impersonal as unfamiliar human groups. One possible explanation is that current AI systems, though increasingly interactive, still lack key qualities of close relationships—such as emotional reciprocity and mutual understanding (J. D. Lee & See, 2004; Tistelgren, 2018). Thus, the level of AI trust may reflect a kind of quasi-social agent trust rather than deeply social trust. Nevertheless, as AI technologies become more emotionally responsive and socially embedded, the boundaries between human and AI relational categories may continue to blur. Some scholars have even raised concerns about emerging forms of “AI addiction”, where individuals prioritize interactions with AI over those with other people—a trend that deserves critical attention from both researchers and society at large (Chen et al., 2025).

Second, consistent with H2, deception experience associated with AI trust and interpersonal trust in culturally distinct ways. Specifically, in the Chinese sample, deception experience negatively associated both AI trust and interpersonal trust, suggesting a generalized erosion of trust rooted in past interpersonal experiences. In contrast, this effect was largely absent in the American sample—except in the case of embodied AI. This pattern suggests that Americans may selectively project emotional experiences onto agents that display human-like features, whereas Chinese individuals tend to generalize relational experiences more broadly, including to less anthropomorphic agents. These cross-cultural differences are in line with prior research showing that East Asians attend more to relational and contextual cues, while Westerners emphasize dispositional attributes of the target (Nisbett et al., 2001). These findings highlight not only that people may apply interpersonal trust schemas to AI interactions (thus supporting the CASA), but also that the extent of this application is shaped by the cultural context of the perceiver.

Third, although H3 was not directly supported—that is, neither social risk nor financial risk significantly mediated the relationship between deception experience and trust in either cultural sample—our exploratory analyses uncovered distinct predictive patterns of risk sensitivity across AI types and cultural contexts. Specifically, in the Chinese sample, social risk had a stronger relationship with trust in embodied AI, whereas financial risk played a greater role in trust in disembodied AI. In contrast, in the American sample, financial risk strongly related with trust in both embodied and disembodied AI. These findings partially support H3 by highlighting the psychological salience of physical embodiment, particularly in the Chinese context. As mentioned in the Introduction, for Chinese users, the physical presence of AI may trigger social expectations and concerns about relationship harmony, reflecting a cultural tendency to treat embodied AI more like a quasi-social agent. In contrast, for American users, AI—whether embodied or disembodied—appears to be treated more as a functional tool, with risk assessments focused on outcome-based and financial consequences, regardless of the AI’s appearance or human-likeness (Haring et al., 2014). In short, Chinese participants are more likely to interpret embodied AI through a relational lens, whereas American participants rely on a utilitarian lens when assessing trust. This illustrates a culturally moderated embodiment effect, where the same physical feature—AI’s embodiment—triggers different cognitive schemas across cultures.

**Figure 3:**
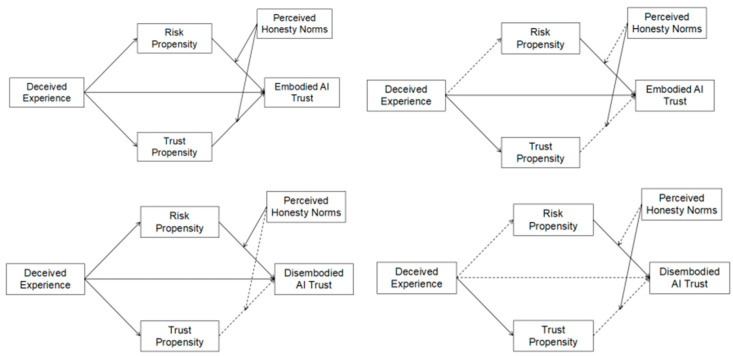
Model results for each sample. Note: Dashed lines indicate non-significant effects, whereas solid lines indicate significant effects. The two panels on the left represent the Chinese sample, and the two panels on the right represent the American sample.

Fourth, the results supported H4a and did not support H4b, as shown in Figure 3. In the Chinese sample, trust propensity played a stronger mediating role than risk propensity, not only in interpersonal trust but also in trust in embodied AI, though not disembodied AI. In contrast, in the American sample, trust propensity only mediated interpersonal trust, and neither dispositional nor risk-based tendencies mediated AI trust significantly. These findings suggest that trust in embodied AI agents—similar with that in humans—is more dispositional in nature, especially among Chinese participants. This aligns with dual-process theories, which differentiate between System 1 (intuitive, affective, and heuristic) and System 2 (deliberative and analytical) processes (Frankish, 2010). That is, while interpersonal trust appears to rely more on System 1 processes—such as personality-based dispositions (Dunning & Fetchenhauer, 2011)—this intuitive trust pathway may extend to embodied AI for Chinese users, reflecting their tendency to treat anthropomorphic agents as social partners. In contrast, for American participants, trust propensity influenced only human–human trust, suggesting that AI is still cognitively processed as a tool rather than intuitively engaged as a social partner. This further illustrates cultural divergence in AI social perception, wherein Chinese individuals may more readily apply interpersonal trust schemas to embodied AI, while Americans maintain a clearer psychological boundary between humans and machines.

Finally, the results supported H5 (Figure 3). In the Chinese sample, perceived honesty norms moderated three key relationships: (1) the effect of trust propensity on interpersonal trust, (2) the effect of risk propensity on disembodied AI trust, and (3) the effects of both trust and risk propensity on embodied AI trust. In the American sample, perceived norms only moderated the effect of trust propensity on (both embodied and disembodied) AI trust, but had no influence on interpersonal trust. These findings point to a cultural divergence in normative sensitivity: in China’s tight cultural context, perceived social norms shape trust decisions across both human and AI targets; in the looser American context, normative influence is selectively applied to AI. One plausible explanation is that, because AI agents remain relatively novel and uncertain, Americans may rely on perceived social expectations as external guidance when evaluating them, whereas interpersonal trust is more firmly grounded in individual dispositions and personal experience. Interestingly, among Chinese participants, perceived honesty norms dynamically moderated the psychological pathway to trusting embodied AI: under low-norm conditions, trust was driven more by risk propensity; under high-norm conditions, trust propensity became the primary driver. This pattern implies that embodied AI occupies a psychologically hybrid space—technically nonhuman yet socially human-like—where trustworthiness depends not only on personal dispositions but also on the prevailing normative expectations. In short, embodied AI may activate both cognitive (risk-based) and affective (propensity-based) routes to trust, with culturally shaped social norms determining which route takes precedence.

This study has several limitations. First, rather than asking participants to classify AI as a social target, we directly measured their trust in each target, which may not fully capture participants’ own perceptions of AI’s social roles. Second, although we theoretically distinguished embodied and disembodied AI based on the presence versus absence of a salient physical form through which interaction occurs, the concrete examples used to illustrate these categories may not have been equally clear to all participants. We intentionally provided a broad range of examples for each AI category in order to convey the conceptual denotation of embodied versus disembodied AI. The broad and heterogeneous range of examples may also have shaped participants’ perceptions by implicitly anchoring them to specific affordances and risk profiles. In addition, trust in embodied and disembodied AI was each assessed using a single-item measure, which may raise psychometric concerns. To partially address these issues, we conducted supplementary analyses controlling for participants’ usage frequency of each AI type and examined overall AI trust using a validated multi-item scale; the main patterns of results remained consistent. Nevertheless, limitations in both operationalization and measurement may have introduced noise and attenuated effect sizes. Future research should therefore employ clearer and more tightly defined stimuli, adopt multi-item or manipulation-based measures of AI trust, and examine more specific AI use contexts in order to strengthen construct validity and further assess the practical applicability of our findings across different AI systems and interaction scenarios. Third, our analyses were correlational in nature; future studies should employ experimental designs to test the causal mechanisms underlying these relationships. Forth, the trust propensity scale used in this study was originally developed for interpersonal settings, which may account for its stronger predictive power for interpersonal trust than for AI trust (Yamagishi et al., 2015). Future research should consider domain-general or AI-specific trust measures to validate and extend this finding.

Furthermore, although some measures were originally developed in English, several constructs showed relatively low internal consistency in the U.S. sample, which may reflect measurement noise and pose limitations for construct validity and cross-cultural comparability. These discrepancies, which were primarily cross-cultural and most pronounced for trust propensity and interpersonal trust, may reflect culturally grounded differences in item interpretation and cognitive processing. Reexamination of the original items, together with the data analysis results, indicated that in the U.S. sample, Item 1 (“Even though I may sometimes suffer the consequences of trusting someone, I still prefer to trust than not to trust others.”) and Item 2 (“I don’t want to do things together with others insofar as there is the slightest possibility that I am the only one to suffer a loss.”) contributed disproportionately to the reduced internal consistency. In our opinion, Item 1 may be interpreted in the Chinese context as a normative orientation toward trust, but in the U.S. context as a strategic judgment about when trust is rational. Similarly, Item 2 may reflect willingness to assume risks for collective cooperation in China, but concerns about fairness or institutional risk in the U.S. context. Such interpretive divergence likely weakens these items’ coherence as indicators of a single latent construct in the U.S. sample. Overall, these findings are consistent with our cultural difference perspective, suggesting systematic cross-cultural variation in how trust is conceptualized and established. Future research should employ refined or expanded multi-item measures to further assess the cross-cultural applicability and interpretive equivalence of trust propensity.

The substantially lower reliability of the Intermediate Group Trust scale in the American sample may reflect cultural differences in how “intermediate” social relationships—such as unfamiliar relatives and ordinary friends—are cognitively structured. In the Chinese context, social relationships are often organized along a relatively continuous relational gradient, in which such targets occupy a recognizable intermediate position between close in-group members and complete strangers, resulting in more coherent trust evaluations. In contrast, in the American context, unfamiliar relatives and ordinary friends may not form a unified psychological category but instead be evaluated according to distinct norms, experiences, or situational cues. This conceptual heterogeneity likely reduces shared variance among items and leads to lower internal consistency. Importantly, this pattern may reflect genuine cultural differences in the organization of social trust rather than mere measurement error, underscoring the need for culturally sensitive operationalizations of intermediate group trust in cross-cultural research.

Fifth, both the Chinese and American samples were recruited via online survey platforms. While this approach facilitates cross-cultural comparison, it may limit the generalizability of the findings to broader national populations. Replication using more diverse sampling methods is therefore warranted. Finally, some key constructs, such as deception experience, were assessed using a relatively small number of items. Although these measures demonstrated acceptable performance in the present study, further psychometric refinement and validation would strengthen confidence in their reliability and construct validity. Additionally, we found that risk propensity positively predicted AI trust, a pattern that diverges from some prior findings (e.g., Ferronato & Bashir, 2020), underscoring the need for future research to further disentangle how risk perception, domain specificity, and individual risk attitudes jointly shape trust in intelligent systems.

In sum, this study advances our understanding of AI trust by revealing both its convergence with and divergence from interpersonal trust across cultural contexts. Our findings show that trust in AI neither mirrors any single category of interpersonal trust nor emerges from entirely distinct processes; instead, it is grounded in psychological mechanisms that partially overlap with, yet remain distinct from, those underlying interpersonal trust. It emerges from a dynamic interplay between past experiences, individual tendencies (e.g., risk and trust propensity), the perceived social affordances of AI (e.g., embodiment), and culturally grounded honesty norms. These results help clarify the theoretical debate between CASA and UAH: although people may apply interpersonal schemas to AI interactions (as CASA suggests), the nature and strength of these applications are moderated by cultural norms and by whether the AI agent is perceived as socially present or functionally instrumental—a distinction emphasized by UAH. This study also highlights that similarity in trust levels does not necessarily imply similarity in underlying mechanisms. For instance, although American participants exhibited AI trust levels comparable to those toward quasi-social agents, the underlying mechanisms of AI trust differed substantially from those of interpersonal trust. Theoretically, our findings support a hybrid perspective, where AI trust is partially social but not fully interpersonal, and its underlying mechanisms vary across cultural settings. Practically, this has implications for the design and deployment of intelligent systems. For instance, calibrating trust in AI may require different strategies in tight versus loose cultures, or in contexts emphasizing emotional closeness versus functional efficiency. Future research should continue to examine how people’s trust in AI generalizes from human relationships, under what conditions these generalizations occur, and how they evolve as AI becomes increasingly embedded in everyday life.

## Figures and Tables

**Table 1:** Characteristics of participants.

Variables	Categories	Chinese Sample		American Sample	
		Frequency	Percentage	Frequency	Percentage
Biological Sex	Male	118	40.8	146	50.7
	Female	171	59.2	142	49.3
	Total	289	100	288	100
Gender Identity	Male	117	40.5	141	49.0
	Female	169	58.5	142	49.3
	Non-binary	2	0.7	5	1.7
	Other	1	0.3	0	0
	Total	289	100	288	100
Age	18–25 years	90	31.1	94	32.7
	26–30 years	102	35.2	92	31.9
	31–50 years	97	33.4	102	35.4
	Total	289	100	288	100
Educational Level	Junior college and below	12	4.1	83	28.8
	Undergraduate	221	76.5	129	44.8
	Master’s degree and above	56	19.4	76	26.4
	Total	289	100	288	100

**Table 2:** Correlations between independent variables and dependent variables.

Variable	*M* (*SD*)		1	2	3	4	5
	Chinese Sample	American Sample					
1 Intimate Group Trust	4.53(0.32)	4.37(0.58)	1	0.46 ***	0.14 *	0.24 ***	0.18 **
2 Intermediate Group Trust	3.21(0.64)	3.21(0.68)	0.69 ***	1	0.44 ***	0.17 **	0.24 ***
3 Distant Group Trust	2.34(0.91)	2.29(1.01)	0.50 ***	0.68 ***	1	0.21 ***	0.18 **
4 Embodied AI Trust	4.05(0.71)	3.46(1.10)	0.29 ***	0.31 ***	0.27 ***	1	0.65 ***
5 Disembodied AI Trust	3.87(0.70)	3.26(1.09)	0.22 ***	0.18 **	0.15 *	0.60 ***	1

Note. Correlation coefficients below diagonal are from Chinese sample and above diagonal are from American sample. * *p* < 0.05, ** *p* < 0.01, *** *p* < 0.001.

**Table 3:** The mediating roles of social risk and financial risk on AI trust and interpersonal trust.

Variables		*b* (*SE*)	
		Chinese Sample	American Sample
DE→SR (a1)		−0.41 ** (0.15)	0.03 (0.02)
DE→FR (a2)		−0.13 (0.11)	0.03 (0.03)
SR→Trust (b1)	SR→Embodied AI Trust	0.03 ** (0.01)	−0.05 (0.10)
	SR→Disembodied AI Trust	0.01 (0.01)	−0.14 (0.11)
	SR→Intimate Group Trust	0.00 (0.01)	−0.12 * (0.06)
	SR→Intermediate Group Trust	0.01 (0.01)	0.00 (0.08)
	SR→Distant Group Trust	0.00 (0.01)	−0.01 (0.11)
FR→Trust (b2)	FR→Embodied AI Trust	0.02 (0.02)	0.41 *** (0.08)
	FR→Disembodied AI Trust	0.05 *** (0.01)	0.31 *** (0.08)
	FR→Intimate Group Trust	0.01 (0.01)	0.11 * (0.05)
	FR→Intermediate Group Trust	0.00 (0.01)	0.04 (0.06)
	FR→Distant Group Trust	0.01 (0.53)	0.14 (0.08)
DE→Trust (c)	DE→Embodied AI Trust	−0.06 ** (0.02)	−0.07 * (0.03)
	DE→Disembodied AI Trust	−0.08 ** (0.02)	−0.04 (0.03)
	DE→Intimate Group Trust	−0.05 *** (0.01)	−0.00 (0.02)
	DE→Intermediate Group Trust	−0.13 *** (0.02)	−0.01 (0.02)
	DE→Distant Group Trust	−0.15 *** (0.03)	−0.03 (0.03)

Note. DE = deception experience, SR = social risk, FR = financial risk. * *p* < 0.05, ** *p* < 0.01, *** *p* < 0.001.

**Table 4:** The mediating roles of risk propensity and trust propensity on AI trust and interpersonal trust.

Variables		*b* (*SE*)	
		Chinese Sample	American Sample
DE→RP (a1)		−0.54 * (0.23)	0.33 (0.21)
DE→TP (a2)		−0.53 *** (0.09)	−0.04 * (0.02)
RP→Trust (b1)	RP→Embodied AI Trust	0.02 * (0.01)	0.04 *** (0.01)
	RP→Disembodied AI Trust	0.03 ** (0.01)	0.02 * (0.01)
	RP→Intimate Group Trust	0.00 (0.00)	0.00 (0.01)
	RP→Intermediate Group Trust	−0.01 (0.01)	0.00 (0.01)
	RP→Distant Group Trust	−0.01 (0.01)	0.01 (0.01)
TP→Trust (b2)	TP→Embodied AI Trust	0.06 *** (0.01)	0.02 (0.09)
	TP→Disembodied AI Trust	0.02 (0.01)	0.04 (0.09)
	TP→Intimate Group Trust	0.04 *** (0.01)	0.23 *** (0.05)
	TP→Intermediate Group Trust	0.09 *** (0.01)	0.29 *** (0.06)
	TP→Distant Group Trust	0.11 *** (0.02)	0.33 *** (0.09)

Note. DE = deception experience, RP = risk propensity, TP = trust propensity. * *p* < 0.05, ** *p* < 0.01, *** *p* < 0.001.

**Table 5:** The moderating role of perceived social norms on AI trust.

Variables	Moderated Mediation	Moderated Mediation Values	*b* (*SE*)	
			Chinese Sample	American Sample
RP→EAI Trust	PHN	Low	0.03 *** (0.01)	0.05 *** (0.01)
		High	0.00 (0.01)	0.03 * (0.01)
RP→DAI Trust		Low	0.04 *** (0.01)	0.03 * (0.01)
		High	0.00 (0.01)	0.01 (0.02)
TP→EAI Trust		Low	0.02 (0.02)	−0.25 * (0.11)
		High	0.08 *** (0.02)	0.14 (0.12)
TP→DAI Trust		Low	−0.00 (0.02)	−0.19 (0.12)
		High	0.03 (0.02)	−0.03 (0.10)

Note. RP = risk propensity, TP = trust propensity, PHN = perceived honesty norms, EAI = embodied AI, DAI = disembodied AI. * *p* < 0.05, *** *p* < 0.001.

**Table 6:** The moderating role of perceived social norms on interpersonal trust.

Variables	Moderated Mediation	Moderated Mediation Values	*b* (*SE*)	
			Chinese Sample	American Sample
RP→Intimate Group Trust		Low	−0.00 (0.00)	0.00 (0.01)
		High	−0.00 (0.00)	−0.01 (0.01)
RP→Intermediate Group Trust		Low	−0.01 (0.01)	0.00 (0.01)
		High	−0.01 (0.01)	−0.00 (0.01)
RP→Distant Group Trust		Low	−0.01 (0.01)	0.02 (0.01)
		High	−0.01 (0.01)	0.01 (0.01)
TP→Intimate Group Trust		Low	0.02 * (0.01)	0.21 *** (0.06)
		High	0.04 *** (0.01)	0.16 * (0.07)
TP→Intermediate Group Trust		Low	0.05 *** (0.02)	0.22 ** (0.08)
		High	0.09 *** (0.02)	0.27 ** (0.08)
TP→Distant Group Trust		Low	0.06 * (0.02)	0.23 * (0.12)
		High	0.12 *** (0.02)	0.46 *** (0.12)

Note. RP = risk propensity, TP = trust propensity, PHN = perceived honesty norms. * *p* < 0.05, ** *p* < 0.01, *** *p* < 0.001.

**Table 7:** Results of hypothesis testing.

Hypothesis	Results	Conclusion
H1a: In the Chinese sample, trust in embodied AI would more closely resemble intimate or intermediate group trust.	not supported	intermediate/distant trust < EAI < intimate trust
H1b: In the American sample, trust in embodied AI would more closely resemble distant group trust.	not supported	intermediate/distant trust < EAI < intimate trust
H2: The association between deception experience and AI trust is expected to vary across cultural contexts, with a more salient negative pattern in the Chinese sample.	supported	Chinese sample: Deception experience was negatively associated with trust in AI (embodied and disembodied). American sample: Deception experience was negatively associated with trust in embodied AI.
H3a: In the Chinese sample, the relationship between deception experience and AI trust is expected to be mediated by risk propensity, with social risk and financial risk representing distinct pathways depending on AI embodiment.	partially supported	Embodied AI trust: Positively associated with social risk, but not with financial risk. Disembodied AI trust: Positively associated with financial risk, but not with social risk.
H3b: In the American sample, financial risk is expected to play a more central mediating role in the relationship between deception experience and AI trust, regardless of AI embodiment.	partially supported	Embodied AI trust: Positively associated with financial risk, but not with social risk. Disembodied AI trust: Same pattern as embodied AI trust.
H4a: In the Chinese sample, individual differences in propensity are expected to play a mediating role in the association between deception experience and AI trust, with trust propensity and risk propensity representing distinct psychological pathways across AI types.	supported	Embodied AI trust: The indirect association via risk propensity was not significant, whereas the indirect association via trust propensity was significant. Disembodied AI trust: Neither the indirect association via risk propensity nor that via trust propensity was significant.
H4b: In the American sample, risk propensity are expected to be more relevant than trust propensity in the association between deception experience and AI trust, regardless of AI embodiment.	not supported	Embodied AI trust: Neither the indirect association via risk propensity nor that via trust propensity was significant. Disembodied AI trust: Same pattern as embodied AI trust.
H5: Perceived honesty norms are expected to moderate the relationship between individual dispositions (risk propensity and trust propensity) and AI trust, such that the relative influence of these dispositions varies across cultural contexts and AI embodiment.	supported	Chinese sample Embodied AI trust: Perceived honesty norms moderated the associations with both risk propensity and trust propensity. Disembodied AI trust: Perceived honesty norms moderated the association with risk propensity, but not with trust propensity. American sample Embodied AI trust: Perceived honesty norms moderated the association with trust propensity, but not with risk propensity. Disembodied AI trust: Same pattern as embodied AI trust.

## Data Availability

The data presented in this study are available on request from the corresponding author.
